# Adaptive grid based multi-objective Cauchy differential evolution for stochastic dynamic economic emission dispatch with wind power uncertainty

**DOI:** 10.1371/journal.pone.0185454

**Published:** 2017-09-29

**Authors:** Huifeng Zhang, Xiaohui Lei, Chao Wang, Dong Yue, Xiangpeng Xie

**Affiliations:** 1 Institute of Advanced Technology, Nanjing University of Posts and Telecommunications, Nanjing, China; 2 China institute of Water Resources and Hydropower Research, Beijing, China; Universita degli Studi della Tuscia, ITALY

## Abstract

Since wind power is integrated into the thermal power operation system, dynamic economic emission dispatch (DEED) has become a new challenge due to its uncertain characteristics. This paper proposes an adaptive grid based multi-objective Cauchy differential evolution (AGB-MOCDE) for solving stochastic DEED with wind power uncertainty. To properly deal with wind power uncertainty, some scenarios are generated to simulate those possible situations by dividing the uncertainty domain into different intervals, the probability of each interval can be calculated using the cumulative distribution function, and a stochastic DEED model can be formulated under different scenarios. For enhancing the optimization efficiency, Cauchy mutation operation is utilized to improve differential evolution by adjusting the population diversity during the population evolution process, and an adaptive grid is constructed for retaining diversity distribution of Pareto front. With consideration of large number of generated scenarios, the reduction mechanism is carried out to decrease the scenarios number with covariance relationships, which can greatly decrease the computational complexity. Moreover, the constraint-handling technique is also utilized to deal with the system load balance while considering transmission loss among thermal units and wind farms, all the constraint limits can be satisfied under the permitted accuracy. After the proposed method is simulated on three test systems, the obtained results reveal that in comparison with other alternatives, the proposed AGB-MOCDE can optimize the DEED problem while handling all constraint limits, and the optimal scheme of stochastic DEED can decrease the conservation of interval optimization, which can provide a more valuable optimal scheme for real-world applications.

## Introduction

Dynamic economic dispatch (DED) plays a key role in power system operation: it assigns optimal output into power generators according to the system load requirement in a certain time period. The main goal of DED is to minimize economic cost while satisfying the output limits, ramp rate limits and transmission loss among different power generators. In comparison to static economic dispatch (ED), DED takes ramp rate limits into consideration, which makes solving DED problems very challenging. Generally, DED can be optimized via the static economic dispatch method by dividing the dispatch period into several intervals [1–3], and many optimization methods have been utilized to solve DED problems, such as evolutionary programming (EP) [4, 5], goal-attainment method [6], quadratic programming (QP) [7], and particle swarm optimization (PSO) [8].

With the increased concern regarding environmental protection, emission pollutants from thermal power generation have received increasing attention. To reduce emission pollutants, especially sulfur oxide and nitric oxide, the Clean Air Act Amendments were ordered to modify the design or operational strategy. However, modifying the design or replacing clean equipment can be quite expensive, and it can be merely taken as a long-term option. The dispatch method for reducing the emission pollutants can be an effective way in the short term and can take emission pollutants into consideration in the DED problem [9]. DED with emission pollutants can be called dynamic economic emission dispatch (DEED), and it is generally considered a multi-objective optimization problem (MOP), in which both the fuel cost and emission rate can be minimized.

Recently, many alternatives have been proposed to solve the DED problem with emission pollutants; generally, three main research directions can be concluded: 1) Treating emission rate as a constraint limit during the optimization process [10]; 2) Converting the MOP into a single-objective optimization problem (SOP) with the weighting technique [11, 12]; 3) Optimizing fuel cost and emission rate simultaneously [13–19]. The first method can only optimize the emission rate to a certain degree, but it cannot minimize the emission rate at extreme effort. In the second method, the exact weight value can be difficult to obtain for real-world applications. The third method can obtain a set of Pareto optimal solutions for different situations without prior knowledge of objective weights; thus, it can be naturally taken as a relatively efficient way to solve the DEED problem. Multi-objective evolutionary algorithms (MOEAs) have also received great attention. Based on the Pareto optimal theory, MOEAs generate Pareto optimal solutions in a single simulation run. In literature [7], the non-dominated sorting genetic algorithm-II (NSGA-II) was used to optimize the DEED problem, which takes emission rate and fuel cost as competing and non-commensurable objectives. In literature [19], multi-objective differential evolution (MODE) was improved to solve the DEED problem and was implemented for a 10-thermal-unit system to verify its efficiency. In literature [20], multi-objective particle swarm optimization (MOPSO) was developed to solve the DEED problem.

Since high penetration of wind power has increasingly become a great challenge to DEED due to its uncertain characteristics [21], great deviation may exist between forecasted wind output and actual wind output, most conventional deterministic methods cannot properly solve DEED with wind power uncertainty. In this paper, scenario technique is utilized on stochastic DEED model to simulate those possible situations caused by wind power uncertainty, and feasible domain of wind power output is divided into several intervals with different probabilities to generate those scenarios. For improving diversity distribution of obtained Pareto front, adaptive grid based multi-objective Cauchy differential evolution is proposed to optimize the stochastic DEED model, Cauchy mutation operation improves differential evolution by adjusting the population diversity, and adaptive grid mechanism retains the diversity distribution of obtained Pareto front. After the proposed AGB-MOCDE is implemented on three test systems, 5-thermal-unit and 10-thermal-unit systems are used to prove the efficiency of AGB-MOCDE and the thermal-wind power system is used to prove its optimization ability to deal with the stochastic DEED problem.

This paper is organized as follows: Section 2 presents the problem formulation of DEED with wind power uncertainty, some basic information is introduced in Section3, and the details of proposed AGB-MOCDE are substantially presented in Section 4. For testifying the efficiency of the proposed method, the implementation details of AGB-MOCDE for stochastic DEED with wind uncertainty are described in Section 5, and the case study and conclusion are presented in Section 6 and Section 7, respectively.

## Problem formulation

Due to the uncertainty of wind power generation, generated scenarios are taken to simulate those possible situations caused by wind power uncertainty [22]. Scenario based technique has been an efficient method for solving stochastic optimization problem, probability distribution of each variable can be represented with a finite set of scenarios, which correspond with realizations of those random variables during the time span [22]. The uncertainty domain of wind output can be divided into several intervals, each situation can be included in one interval, and the probability of each interval can be calculated combined with probability density function of wind power generation. The expected value of fuel cost and emission rate can be taken as two objectives, and the conventional DEED model can be developed into a stochastic DEED combined with generated scenarios.

### 1. Economic objective

Since it doesn’t exist economic cost for wind power generation in thermal-wind power system, fuel cost can be taken as the main economic cost. The fuel cost can be expressed by the summation of quadratic function and sinusoidal function of thermal output, which is caused by the sudden opening of the intake valve of a steam turbine. Generally, economic cost with the valve-point effect can be properly described as follows [23]:

$$f_{1}=\sum_{t=1}^{T}\sum_{i=1}^{N_{c}}\left(a_{i}P_{ci,t}^{2}+b_{i}P_{ci,t}+c_{i}+\left|d_{i}\sin(h_{i}(P_{ci,t}-P_{ci,\min}))\right|\right)$$ (1)

where *T* represents the total time period length, *N*_*c*_ is number of thermal units, *P*_*ci*,*t*_ represents the output of the *i*th thermal unit in the *t*th time period, *P*_*ci*,min_ is the minimum output of the *i*th thermal unit, and *a*_*i*_,*b*_*i*_,*c*_*i*_,*d*_*i*_,*h*_*i*_ are the cost coefficients of fuel cost at the *i*th thermal unit. In the stochastic model, combined with the probability density of wind power output, the expected value of economic cost can be expressed as follows:

$$F_{1}=E(f_{1})=\sum_{s=1}^{N_{s}}\rho_{s}\sum_{t=1}^{T}\sum_{i=1}^{N_{c}}\left(a_{i}P_{ci,t}^{s}{}^{2}+b_{i}P_{ci,t}^{s}+c_{i}+\left|d_{i}\sin(h_{i}(P_{ci,t}^{s}-P_{ci,\min}))\right|\right)$$ (2)

where *E*(∙) represents the expected value of economic cost, *ρ*_*s*_ is the probability that the *s*th scenario occurs, $P_{ci,t}^{s}$ is the output of the *i*th thermal unit in the *t*th time period in the *s*th scenario, and *N*_*s*_ is the number of generated scenarios.

### 2. Emission objective

The emission pollutant of thermal units represents another objective in the DEED problem, it mainly consists of sulfur dioxide and nitrogen oxides, and can be generally expressed with thermal output. Generally, emission pollutant caused by thermal units can be described as a combination of polynomial and exponential terms, which can be formulated as [24]:

$$f_{2}=\sum_{t=1}^{T}\sum_{i=1}^{N_{c}}\left[\alpha_{i}+\beta_{i}P_{ci,t}+\gamma_{i}P_{ci,t}^{2}+\zeta_{i}\exp(\lambda_{i}P_{ci,t})\right]$$ (3)

where *α*_*i*_,*β*_*i*_,*γ*_*i*_,*ζ*_*i*_,*λ*_*i*_ are the coefficients of emission rate at the *i*th thermal unit. Similarly, the expected value of the emission rate can be expressed as follows:

$$F_{2}=E(f_{2})=\sum_{s=1}^{N_{s}}\rho_{s}\sum_{t=1}^{T}\sum_{i=1}^{N_{c}}\left[\alpha_{i}+\beta_{i}P_{ci,t}^{s}+\gamma_{i}P_{ci,t}^{s}{}^{2}+\zeta_{i}\exp(\lambda_{i}P_{ci,t}^{s})\right]$$ (4)

### 3. Constraints

(1) Power balance constraint [6, 25]

$$\sum_{i=1}^{N_{c}}P_{ci,t}^{s}+\sum_{j=1}^{N_{w}}P_{wj,t}^{s}=P_{load,t}^{s}+P_{loss,t}^{s}$$ (5)

where $P_{wj,t}^{s}$ is the output of the *j*th wind farm in the *t*th time period in the *s*th scenario, $P_{load,t}^{s},P_{loss,t}^{s}$ are system load requirement and transmission loss, respectively, in the *t*th time period in the *s*th scenario, and *N*_*w*_ is the number of wind farms. The transmission loss among different power generators is taken into consideration, which can be generally expressed as follows:

$$P_{loss,t}=\sum_{i=1}^{N_{c}+N_{w}}\sum_{j=1}^{N_{c}+N_{w}}{ℜ}_{i,t}^{s}B_{ij}{ℜ}_{j,t}^{s}+\sum_{i=1}^{N_{c}+N_{w}}B_{0i}{ℜ}_{i,t}^{s}+B_{00}$$ (6)

where *B*_*ij*_,*B*_0*i*_,*B*_00_ are the coefficients of transmission loss and ${ℜ}_{i,t}^{s}$ is the output of the *i*th power generator in the *t*th time period in the *s*th scenario.

(2) Output limits

$$P_{ci,\min}\leq P_{ci,t}^{s}\leq P_{ci,\max}$$ (7)

where *P*_*ci*,max_ is the maximum output of the *i*th thermal unit.

(3) Ramp rate limits

$$DR_{ci}\leq P_{ci,t}^{s}-P_{ci,t-1}^{s}\leq UR_{ci}$$ (8)

where *DR*_*ci*_,*UR*_*ci*_ are the down-ramp and up-ramp rates, respectively, of the *i*th thermal unit.

(4) The relationship between wind output and wind speed

The wind power output is closely related to wind speed; its relationship can be generally described as follows [26]:

$$P_{wj,t}^{s}=\{\begin{array}{cc} 0 & v_{j,t}^{s}<v_{j,in}\;or\;v_{j}\geq v_{j,out}\\ P_{wj,\max}*\frac{v_{j,t}^{s}-v_{j,in}}{v_{j,rate}-v_{j,in}} & v_{j,in}\leq v_{j}\leq v_{j,rate}\\ P_{wj,\max} & v_{j,rate}\leq v_{j}\leq v_{j,out} \end{array}$$ (9)

where $v_{j,t}^{s}$ is the wind speed of the *j*th wind farm in the *t*th time period in the *s*th scenario, *v*_*j*,*rate*_ is the rated wind speed of the *j*th wind farm, *v*_*j*,*in*_,*v*_*j*,*out*_ are the cut-in and cut-out wind speed, respectively, at the *j*th wind farm, and *P*_*wj*,max_ is the maximum output at the *j*th wind farm.

## Related works

### 1. The description of the multi-objective optimization problem

The MOP generally has two or more competing objectives and cannot generate a single optimal solution; rather, it generates a set of non-dominated solutions in a single simulation run. In comparison to single-objective optimization, the fitness function cannot properly describe the dominance relationship among those individuals in an evolutionary population. For a given MOP, the MOP can generally be described as follows:

$$\left\{ \begin{array}{l} \min F(X)=\{\min f_{1},\min f_{2},\ldots ,\min f_{m}\}\\ s.t.\;g_{j}(X)\leq 0\;j=1,2,\ldots ,S_{g}\\ h_{k}(X)=0\;k=1,2,\ldots ,S_{h} \end{array}\right.$$ (10)

where *F*(∙) is the objective vector, which contains *m* objective functions, *g*_*j*_(∙) is the *j* th inequality constraint, *h*_*k*_(∙) is the *k* th equality constraint, and *S*_*g*_,*S*_*h*_ are the number of inequality and equality constraints, respectively.

In comparison to single-objective optimization, multi-objective optimization utilizes the Pareto dominance relationship to decide which individual should be better. Because the objectives in the MOP are generally in conflict with each other, the relationship among different individuals cannot be easily described with a certain order. In the MOP, the Pareto dominance relationship with a partial order is utilized to describe the relationship among those individuals; the dominance order and Pareto optimal solution can be properly obtained using this dominance relationship.

**Definition 1**: Assume two feasible solutions *x*_1_,*x*_2_; the relationship between them exists as:

$$f_{i}(x_{1})\leq f_{i}(x_{2})\;,i=1,2,\ldots ,m$$ (11)

The Pareto dominance relationship between *x*_1_ and *x*_2_ is that *x*_1_ dominates *x*_2_ and that *x*_1_ has higher Pareto dominance order than *x*_2_, which can be denoted as *x*_1_ ≻ *x*_2_.

**Definition 2**: Assume that no solutions can dominate x^*^ in the solution set; x^*^ is called the non-dominated solution or Pareto optimal solution in the solution set, which can be described as: ∀*i* = 1,2,…,*n*,¬*x*_*i*_ ≻ x^*^.

**Definition 3**: The set of objective vectors obtained from Pareto optimal solutions is called the Pareto front, which can be described as follows:

$$PF=\{F(x^{*})|\forall i=1,2,\ldots ,n,\neg x_{i}≻x^{*}\}$$ (12)

### 2. Outline of differential evolution (DE)

DE is widely known as a simple yet powerful optimization algorithm due to its smaller number of parameters and population-based evolutionary strategy. The basic operations of DE include mutation, crossover and selection, the details about DE has been presented in literature [19]. Combined with the above three basic operations, DE can guide individual vectors to move into the optimal solution. However, conventional DE cannot avoid a premature problem as other evolutionary algorithms can due to its fixed scaling parameter; it is apt to fall into local optima, especially for multi-modal functions. Here, the Cauchy mutation method is utilized to improve the differential evolution by checking population diversity, which can guide population evolution adaptively.

## Interactive fuzzy satisfying-based adaptive grid-based multi-objective Cauchy differential evolution

For properly optimizing stochastic DEED problem, the AGB-MOCDE algorithm is proposed to improve the optimization efficiency as follows: (1) For avoiding the premature problem, adaptive Cauchy mutation operator is utilized to search global optimal solution by properly controlling population diversity during evolution process; (2) The adaptive grid based diversity maintenance strategy is proposed to properly control the diversity distribution, it makes obtained Pareto-optimal solutions evenly distributed, which can be convenient for decision-makers; (3) Interactive fuzzy satisfying method is utilized as a selection mechanism to screen the best optimal solution from those obtained nominated solutions, and the selected solution can be taken as the best scheme for DEED problem.

### 1. Adaptive Cauchy mutation-based multi-objective differential evolution

The essential reason for the premature problem is that the diversity of the population decreases during the evolution process [27]. To avoid the premature problem, population diversity can be checked using the convergence metric *C*(*P*^(*g*)^), which denotes convergence progress as the number of generations increases [28]. If the convergence metric *C*(*P*^(*g*)^) does not change obviously after *h* generations, which also means that ∇_*CP*_ is smaller than a threshold *ξ*_*CP*_ ∈ [0.01,0.1], where ∇_*CP*_ is defined as |*C*(*P*^(*g*)^)−*C*(*P*^(*g*−*h*)^)|/*C*(*P*^(*g*)^), check the population diversity. If the population diversity is less than a threshold *ε*, adaptive Cauchy mutation is carried out to update the current individuals and is expressed as follows:

$$x_{i,j,G}=x_{i,j,G}\cdot (1+\eta \cdot C(0,1)),\;if\;diversity\;(j)<\varepsilon \;and\;rand()<0.5$$ (13)

where *C*(0,1) is a standard Cauchy variable, *η* ∈ [0.1,0.5] is a coefficient of Cauchy mutation, *ε* is the diversity threshold, and *diversity*(*j*) is the diversity value on the *j*th dimension, which is described as follows:

$$diversity(j)=\sqrt{1/N_{Q}\sum_{i=1}^{N_{Q}}{\left(\frac{x_{i,j,G}-{\bar{x}}_{j,G}}{u_{j}-l_{j}}\right)}^{2}}$$ (14)

where *N*_*Q*_ is the size of the archive set, ${\bar{x}}_{j,G}$ is the average value of the *j*th dimension variables, and *u*_*j*_,*l*_*j*_ are the upper and lower bounds, respectively, of the *j*th dimension variables. According to formulation (13), parameter *η* can adjust the search scale of the Cauchy mutation operation; the linear formulation in literature [29] is taken to control the mutation operation as population evolution proceeds. The linear formulation is presented as follows:

$$\eta =0.5-0.4*G/G_{\max}$$ (15)

where *G*_max_ is the maximum generation number. Combined with the above adaptive mutation operation, the differential evolution process can be properly controlled using population diversity, and it can adjust the search scale using adaptive scaling parameters, which can avoid the premature problem as population evolution proceeds.

### 2. Adaptive grid-based diversity maintenance strategy

Since archive set has a certain size, diversity maintenance strategy is needed when archive set is full. Here, an adaptive grid-based diversity maintenance strategy is proposed to improve diversity distribution of optimal individuals in each generation. Two-dimensional coordinate is divided into several small boxes, which are taken to measure the diversity distribution of Pareto optimal front.

#### 3.1 Grid setting

In the current archive set, the extreme values of optimal individuals can be found, it labels minimum value and maximum value of objective function *F*_1_ as $F_{1}^{\min},F_{1}^{\max}$, and minimum value and maximum value of objective function *F*_2_ as $F_{2}^{\min},F_{2}^{\max}$, and all those optimal individuals can be shown in sorted order in a two-dimensional coordinate. The grid division depends on the number of non-dominated solutions, and each square can be obtained:

$$\left\{ \begin{array}{l} \delta_{F_{1}}=\frac{\left|F_{1}^{\max}-F_{1}^{\min}\right|}{N_{Q}}\\ \delta_{F_{2}}=\frac{\left|F_{2}^{\max}-F_{2}^{\min}\right|}{N_{Q}} \end{array}\right.$$ (16)

The $\delta_{F_{1}},\delta_{F_{2}}$ represents the square length in *F*_1_,*F*_2_ objective direction, and feasible region can be divided into *N*_*Q*_ × *N*_*Q*_ pieces. Those optimal individuals scattered on those pieces need to keep certain distribution characteristics for ensuring diversity distribution of Pareto front. For each optimal individual, it satisfies:

$$\left(F_{1}^{*(i)},F_{2}^{*(i)})\in \underset{j=1..N_{Q}}{\cup }\{[F_{1}^{\min}+(j-3/2)\delta_{F_{1}},F_{1}^{\min}+(j-1/2)\delta_{F_{1}}]\cap [F_{2}^{\max}+(1/2-j)\delta_{F_{2}},F_{2}^{\max}+(3/2-j)\delta_{F_{2}}]\right\}$$ (17)

It is assumed that all optimal individuals are sorted by objective *F*_1_ in ascending order $\left\{i_{k_{1}},i_{k_{2}},\ldots ,i_{k_{N_{Q}}}\right\}$, grid setting for $i_{k_{j}}$th individual must satisfy:

$$\left(F_{1}^{*(j)},F_{2}^{*(j)})\in [F_{1}^{\min}+(i_{k_{j}}-3/2)\delta_{F_{1}},F_{1}^{\min}+(i_{k_{j}}-1/2)\delta_{F_{1}}]\cap [F_{2}^{\max}+(1/2-i_{k_{j}})\delta_{F_{2}},F_{2}^{\max}+(3/2-i_{k_{j}})\delta_{F_{2}}\right]$$ (18)

#### 3.2 Grid based diversity maintenance mechanism of Pareto front

**Fig 1:**
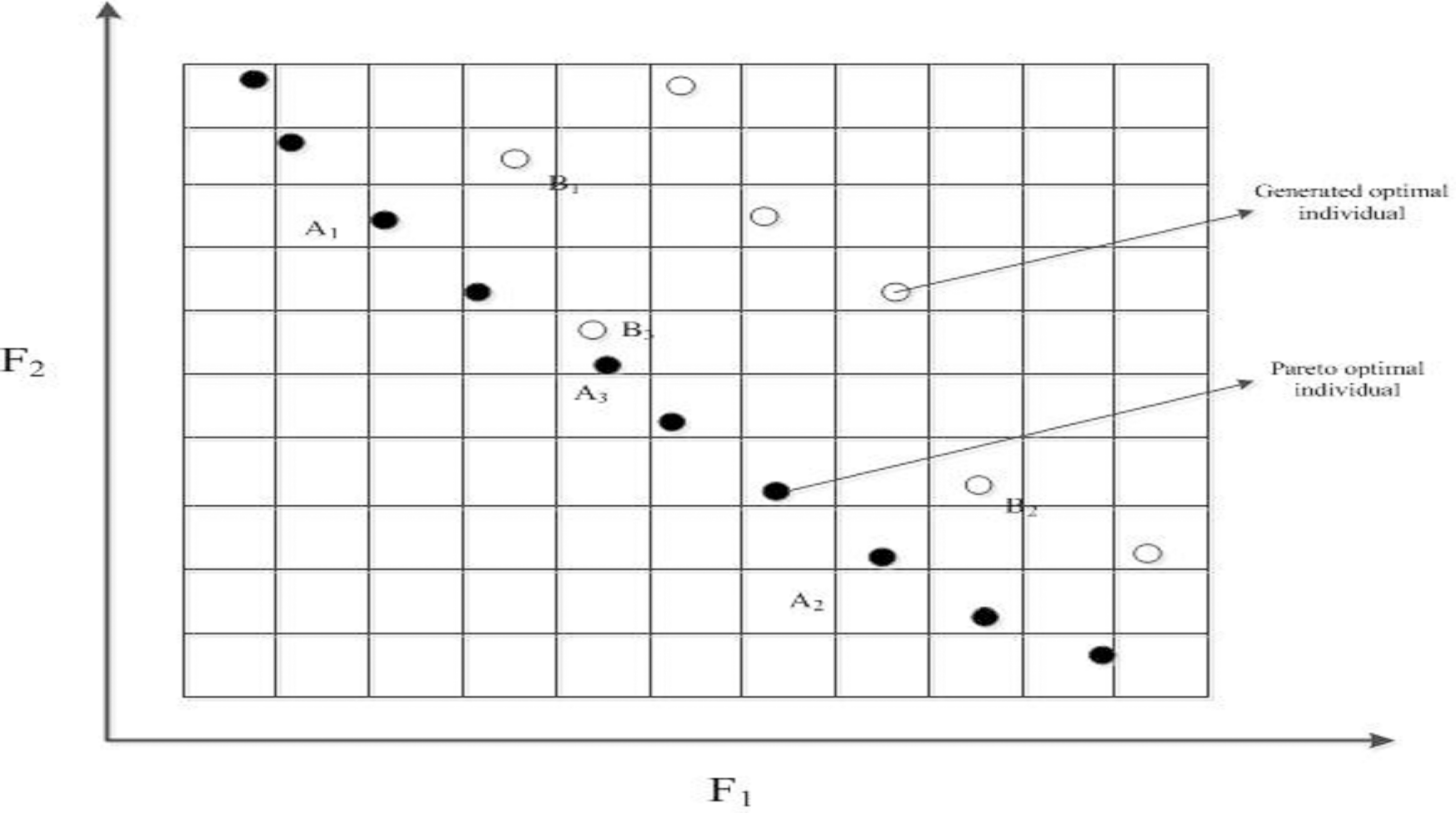
Grid-based diversity maintenance mechanism of Pareto front.

Since archive set has a certain size, truncation mechanism must be taken to keep the elitism when the number of Pareto optimal individuals exceed the size of archive set. On the basis of above grid setting, the diversity distribution is taken to justify whether newly generated optimal solution is added into archive set when archive set is full. If generated optimal individuals *B*_1_,*B*_2_ are in the grids dominated by those individuals *A*_1_,*A*_2_ in archive set as it is shown in **Fig 1**, the newly generated solution can’t be added into the archive set.

If newly generated individual *B*_3_ and Pareto optimal individual *A*_3_ are in the same grid, diversity distribution of Pareto optimal front is taken to judge which one should be replaced by the other one. The main procedures are presented as follows:

**Step.1**: Add the newly generated individual into archive set, calculate the number of individuals in each grid $\left\{k_{1},k_{2},k_{3},\ldots ,k_{N_{Q}}\right\}$, which satisfies $\sum_{i=1}^{N_{Q}}k_{i}=N_{Q}+1$ and *k*_*i*_ ∈ *N*.
**Step.2**: Find the $k_{\max}=\underset{i=1..N_{Q}}{\max}(k_{i})$, and if there are *N*_*grid*_ grids that contain *k*_max_ individuals, and select all individuals $\left\{L_{1},L_{2},\ldots ,L_{N_{grid}*k_{\max}}\right\}$ in these grids.
**Step 3**: Calculate density degree of these individuals with following metrics:

$$\left\{ \begin{array}{l} Den(L_{i})=\sum_{j\neq L_{i},j\in Q}\frac{1}{dis(L_{i},j)}\\ dis(L_{i},j)=\sqrt{{\left(F_{1}^{L_{i}}-F_{1}^{j}\right)}^{2}+{\left(F_{2}^{L_{i}}-F_{2}^{j}\right)}^{2}} \end{array}\right.$$ (19)

**Step.4**: Select the individual that has the largest density, and delete this individual from the archive set, then archive set can be properly maintained to certain extent. Especially when newly generated individual has better minimum value in objective in *F*_1_ or *F*_2_, this new individual can be directly added into archive set and delete one individual that has the largest density.

### 3. Interactive fuzzy satisfying method as a selection mechanism

Due to the uncertain features of wind power and a decision-maker’s judgment, objective values can be naturally considered fuzzy or imprecise, which brings a challenge to the conventional selection mechanism in multi-objective optimization. This paper utilizes the interactive fuzzy satisfying method to decide which individual vector in the archive set is the best optimal individual vector. The satisfaction of the individual vector in the archive set can be described using membership functions, which can be expressed as follows [30, 31]:

$$\mu_{fq}(X)=\{\begin{array}{cc} 0, & if\;f_{q}(X)\geq f_{q}^{\max}\\ \frac{f_{q}^{\max}-f_{q}(X)}{f_{q}^{\max}-f_{q}^{\min}}, & if\;f_{q}^{\min}\leq f_{q}(X)\leq f_{q}^{\max}\\ 1, & if\;f_{q}(X)\leq f_{q}^{\min} \end{array}$$ (20)

where $f_{q}^{\min},f_{q}^{\max}$ are the minimum and maximum value, respectively, of the *q*th objective function. In the decision-making process, desirable levels of the membership function or reference membership value *μ*_*rq*_ need to be specified; the best optimal solution can be selected by solving the mini-max problem, as follows:

$$\underset{X\in Q}{\min}[\underset{q=1,2}{\max}\left|\mu_{fq}(X)-\mu_{rq}\right|]$$ (21)

In the selection operation, formulation (24) is taken to decide which individual is the best individual that satisfies the decision maker’s requirement. The fuzzy satisfying method takes the uncertainty of environmental circumstances and subjective consciousness into consideration, being able to obtain the best optimal solution, which can be a better fit for real-world applications.

## Implementation of fuzzy satisfying method-based multi-objective differential evolution for a stochastic DEED problem

Because DEED with wind power uncertainty has various coupled constraints with randomness characteristics, the implementation of the proposed algorithm has a great impact on the efficiency of solving the DEED problem. The maximum and minimum output of wind power can be obtained according to its probability density function (PDF), and the output domain can be equally divided into several intervals. Simultaneously, the system load balance constraint with transmission loss connects the thermal units and wind farms together; its constraint handling technique plays an important role in solving the DEED problem. Here, the coupled constraint-handling technique simplifies the system load balance constraint, which ensures that the proposed MODE can be properly implemented for the DEED problem with wind power uncertainty.

### 1. The solution-encoding strategy

In the DEED problem, a set of thermal outputs with *N*_*s*_ scenarios is taken as the decision variable. In each scenario, there are *T* scheduling periods on the schedule horizon, and the output of *N*_*c*_ thermal units needs to be properly assigned in each scheduling period; the decision variable can be encoded as follows:

$$X=\left[\begin{array}{cccccccccc} P_{c1,0}^{1} & \cdots & P_{cN_{c},0}^{1} & P_{c1,0}^{2} & \cdots & P_{cN_{c},0}^{2} & \cdots & P_{c1,0}^{N_{s}} & \cdots & P_{cN_{c},0}^{N_{s}}\\ P_{c1,1}^{1} & \cdots & P_{cN_{c},1}^{1} & P_{c1,1}^{2} & \cdots & P_{cN_{c},1}^{2} & \cdots & P_{c1,1}^{N_{s}} & \cdots & P_{cN_{c},1}^{N_{s}}\\ \vdots & \cdots & \vdots & \vdots & \cdots & \vdots & \cdots & \vdots & \cdots & \vdots \\ P_{c1,T-1}^{1} & \cdots & P_{cN_{c},T-1}^{1} & P_{c1,T-1}^{2} & \cdots & P_{cN_{c},T-1}^{2} & \cdots & P_{c1,T-1}^{N_{s}} & \cdots & P_{cN_{c},T-1}^{N_{s}} \end{array}\right]$$ (22)

### 2. The probability of the output interval under wind power uncertainty

The integration of wind power generation brings strong randomness into the DEED problem, which presents a great challenge for optimization methods. The randomness characteristics of wind power generation need to be analyzed with its probability density function and cumulative distribution function, which can be generally described based on the wind speed in literature [32]:

$$\left\{ \begin{array}{l} f(v_{j})=(k/c){\left(v_{j}/c\right)}^{k-1}\exp(-{\left(v_{j}/c\right)}^{k}),\;v_{j}\geq 0\\ F_{w}(v_{j})=1-\exp(-{\left(v_{j}/c\right)}^{k}),\;v_{j}\geq 0 \end{array}\right.$$ (23)

where *v*_*j*_ is the wind speed of the *j*th wind farm; *k*,*c* are the scaling parameters. Combined with the relationship presented in formula (9), the cumulative distribution function for wind power output can be obtained as:

$$F_{w}(P_{wj})=1-\exp\{-{\left[(1+\frac{v_{j,rate}-v_{j,in}}{v_{j,in}P_{wj\max}}P_{wj})\frac{v_{j,in}}{c}\right]}^{k}\}+\exp[-{\left(v_{j,out}/c\right)}^{k}],\;0\leq P_{wj}<P_{wj\max}$$ (24)

For a certain output interval [*l*_*j*_,*u*_*j*_], the probability of the output in this interval can be calculated as:

$$\mathrm{Pr}ob_{wj,t}^{s}=\mathrm{Pr}ob(l_{j}\leq P_{wj,t}^{s}\leq u_{j})=F_{w}(u_{j})-F(l_{j})$$ (25)

### 3. The division interval of the wind power output

**Fig 2:**
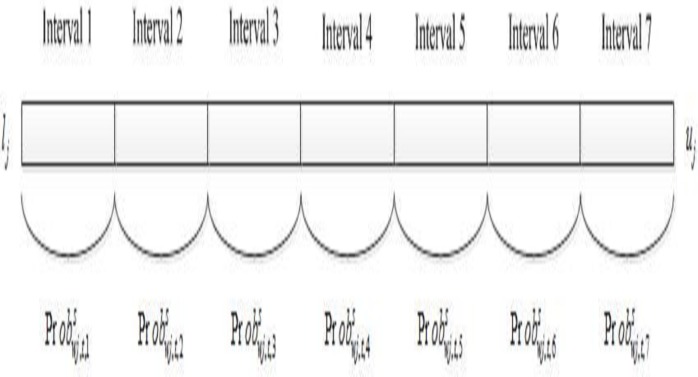
Interval division of wind power generation.

Regarding the randomness of wind power generation, the feasible domain of wind power output can be divided into seven intervals, with different probabilities for each interval. The feasible domain can be equally divided, and the probability of each interval $\mathrm{Pr}ob_{wj,t,\mathrm{int}erval}^{s}$ can be properly calculated using the method in Section 4.2. For a given feasible domain [*l*_*j*_,*u*_*j*_], the interval division of wind power generation can be obtained as in **Fig 2**.

Because scenarios of the thermal wind-power system simulate those possible wind outputs during the entire time period, the binary parameter $I_{wj,t,\mathrm{int}erval}^{s}$ of wind power generation exists in each interval; the interval index int*erval* is selected when int*erval* = 1; otherwise, the interval index int*erval* is not selected. In each generated scenario, the probability of the generated scenario can be described by the Bayesian probability formulation:

$$\rho_{s}=\frac{\sum_{j=1}^{N_{w}}\left(\sum_{\mathrm{int}erval=1}^{7}\left(I_{wj,t,\mathrm{int}erval}^{s}\cdot \mathrm{Pr}ob_{wj,t,\mathrm{int}erval}^{s}\right)\right)}{\sum_{s=1}^{N_{s}}\sum_{j=1}^{N_{w}}\left(\sum_{\mathrm{int}erval=1}^{7}\left(I_{wj,t,\mathrm{int}erval}^{s}\cdot \mathrm{Pr}ob_{wj,t,\mathrm{int}erval}^{s}\right)\right)}$$ (26)

where $I_{wj,t,\mathrm{int}erval}^{s}$ represents the binary parameter of the output interval of wind power generation in the *j*th wind farm in the *t*th time period in the *s*th scenario.

### 4. The reduction of scenarios number

Since a lot of scenarios need to be generated to simulate the power generation process in the power system operation, it makes large dimensionality for high computational complexity. Here, a reduction of scenarios method is proposed to screen out those similar scenarios for simulating more power generation process with less scenarios, a covariance based method is utilized to find covariance between every two scenarios, and screen out one similar scenario with minimal covariance, which can retain the efficiency of the scenario based method. The procedures of covariance based scenarios reduction method can be presented as follows:

**Step 1:** Initialize the reduced scenario set *Scen*_*reduced*_ = *Scen*, and the size of reduced scenarios set is $N_{s}^{reduced}=N_{s}$, required scenarios number is $N_{s}^{need}(N_{s}^{need}<N_{s})$, *S*_*i*_ represents the *i*th scenario in *Scen*_*reduced*_, go to **Step 2**;
**Step 2**: Define the covariance value set $Cov=\{Cov_{ij}|i,j\in 1,2\ldots N_{s}^{reduced}\}$, which can be considered as the covariance matrix. Calculate the covariance between every two scenarios (*i*,*j* ∈ 1,2…*N*_*s*_), and covariance value set can be initialized with *Cov*_*ij*_ = *Co*var*iance*(*S*_*i*_,*S*_*j*_), go to **Step 3**;
**Step 3**: Calculate the eigenvalues of covariance matrix *Cov*, and it can obtain $N_{s}^{reduced}$ eigenvalues, and go to **Step 4**;
**Step 4**: Select the maximum eigenvalue $Eigen_{\max}^{N_{s}}$ and eigenvector $EigenV_{\max}^{N_{s}}$, and compare the eigenvector with each scenario, and find two most similar scenarios $S_{eigen\max1}^{N_{s}}$ and $S_{eigen\max2}^{N_{s}}$, and delete one $S_{eigen\max2}^{N_{s}}$, and go to **Step 5**;
**Step 5**: Then $Scen_{reduced}=Scen_{reduced}-S_{eigen\max2}^{N_{s}}$, $N_{s}^{reduced}=N_{s}^{reduced}-1$, and *ρ*_*eigen*,max1_ = *ρ*_*eigen*,max1_ + *ρ*_*eigen*,max2_; and go to **Step 6**;
**Step 6**: If $N_{s}^{reduced}>N_{s}^{need}$, go to **Step 2**. Otherwise, complete the scenarios reduction procedures and exit.

### 5. The constraint-handling method for system load balance

The proper constraint-handling technique can improve the efficiency of the proposed algorithm, especially when the system load balance constraint is properly handled. Here, transmission loss among different power generators is taken into consideration, which increases the difficulty of handling the system load balance constraint. A constraint technique is utilized to decrease the deviation of the system load balance constraint, which can be calculated as follows:

$$\Omega_{p,t}=\sum_{i=1}^{N_{c}}P_{ci,t}+\sum_{j=1}^{N_{w}}P_{wj,t}-L_{load,t}-L_{loss,t}$$ (27)

Replace transmission loss *L*_*loss*,*t*_ with formulation (6); then, the variation of the deviation can be found as follows:

$$\Delta \Omega_{p,t}=\sum_{i=1}^{N_{c}}(1-B_{0i})\Delta P_{ci,t}-2\sum_{i=1}^{N_{c}}\sum_{j=1}^{N_{c}}B_{ij}P_{ci,t}\Delta P_{cj,t}-2\sum_{i=1}^{N_{c}}\sum_{j=1}^{N_{w}}B_{ij}P_{wj,t}\Delta P_{ci,t}$$ (28)

All thermal output is adjusted with the same increment of deviation, which means ℵ = Δ*P*_*it*_ = Δ*P*_*jt*_(*i* ≠ *j*); the increment of deviation can be obtained as follows:

$$ℵ=\frac{\Delta \Omega_{p,t}}{\sum_{i=1}^{N_{c}}\left(1-B_{0i}\right)-2\sum_{i=1}^{N_{c}}\sum_{j=1}^{N_{c}}B_{ij}P_{ci,t}-2\sum_{i=1}^{N_{c}}\sum_{j=1}^{N_{w}}B_{ij}P_{wj,t}}$$ (29)

According to the above increment of deviation, thermal output can be properly adjusted using the coupled constraint-handling technique in literature [19]. The system load balance constraint can be properly handled using the coarse adjustment and fine tuning technique, and the total constraint violation is utilized to ensure the feasibility of those individuals in the evolutionary population.

### 6. The flowchart of implementation for the DEED problem with wind power uncertainty

The DEED with wind power uncertainty is a complex-coupled optimization problem; all the procedures must be substantial and intact. The interval of wind power output can be equally divided into seven intervals in each scenario, and the probability of each interval can be calculated by solving formulation (25) using the probability density function; the obtained probability of each interval can be input into the DEED model. Then, the proposed MODE with the fuzzy satisfying method can be used to solve the DEED model with the constraint handling technique. The procedure details can be described as follows:

**Step 1**: According to the wind output domain of each wind farm in each time period, generate *N*_*s*_ scenarios in the thermal wind-power system, divide the uncertainty domain of each wind output into seven intervals, calculate the probability of each interval, and input it into the DEED model; go to **Step 2**;
**Step 2**: Combined with DEED under wind power uncertainty, take thermal output as the decision variable, initialize all the parameters and the evolutionary population, and set the generation number *g* = 0; go to **Step 3**;
**Step 3**: Check the population diversity *diversity* (*j*); if *diversity* (*j*) < *ε*, adaptive Cauchy mutation is taken to update the individual vector; then, go to **Step 4**;
**Step 4**: Check the feasibility of individuals in the evolutionary population and use the constraint-handling technique to deal with those infeasible individuals; go to **Step 5**;
**Step 5**: Apply the crossover and selection operations to the evolutionary population, screen out those non-dominated individuals and store them into the archive set via the archive retention strategy; go to **Step 6**;
**Step 6**: If *g* < *g*_max_, *g* = *g* + 1; go to **Step 3**; otherwise, output all the non-dominated individuals in the archive set and go to **Step 7**;
**Step 7**: For a given reference membership value *μ*_*rq*_, select the optimal individual that has the best satisfying value from the archive set; the selected optimal individual vector is the optimal solution of the DEED problem.

## Experimental results and discussion

In this section, the proposed AGB-MOCDE is implemented for three test systems, the whole scheduling time period is 24h with each hour an interval. Test system 1 consists of five thermal units with minimizing fuel cost and emission rate, and non-linear power generation loss is taken into consideration combing with valve point effect. Test system 2 consists of 10 thermal units with the valve-point effect, considering transmission loss among the thermal units, it mainly demonstrates the efficiency of the proposed multi-objective optimization method, and scenario and interval division methods are not required in this test system. Test system 3 consists of 10 thermal units and 3 wind farms with the valve-point effect, considering transmission loss among the thermal units and wind farms, it does not merely verify the efficiency of the proposed multi-objective optimization method, and it also shows its ability to deal with wind power uncertainty.

### 1. Test system 1

**Fig 3:**
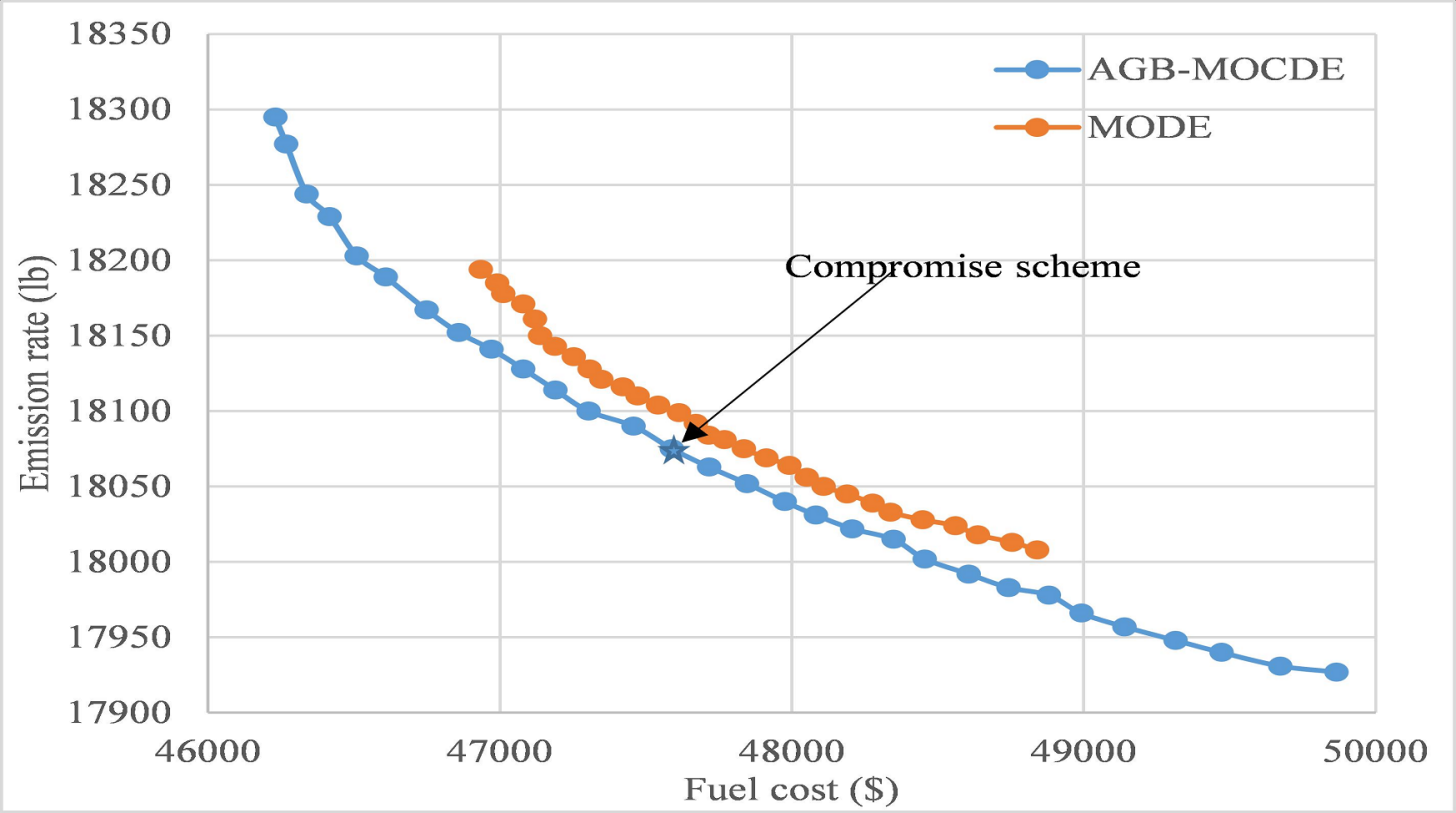
The obtained Pareto front of AGB-MOCDE and MODE.

The main goal of this test system is to properly assign the output of five thermal units to minimize fuel cost and emission rate simultaneously, all data details about five thermal units are presented in literature [33]. In **Fig 3**, 30 non-dominated solutions are produced by AGB-MOCDE method, and are shown with obtained schemes by MODE [34], which is a Pareto dominance based algorithm with DE/rand/1/bin strategy. It can be seen that all non-dominated solutions by AGB-MOCDE are more evenly distributed than that in MODE due to its adaptive grid-based mechanism, and has better convergence ability than MODE.

For further analysis on efficiency of obtained Pareto front, compromise schemes represents those obtained Pareto fronts with calculating fuzzy satisfying membership of those non-dominated schemes, which are presented in **Table 1**. According to formula (21), if it is assumed that reference membership value *μ*_*rq*_ is 0.75, scheme (14) can be selected as compromise scheme by AGB-MOCDE and scheme (16) is taken as compromise scheme by MODE.

**Table 1:** The memberships of non-dominated schemes by AGB-MOCDE and MODE for test system 1.

Scheme	AGB-MOCDE		MODE		Scheme	AGB-MOCDE		MODE	
	Cost	Emission	Cost	Emission		Cost	Emission	Cost	Emission
**1**	1	0	1	0	**16**	0.555556	0.660326	0.590981	0.591398
**2**	0.989549	0.048913	0.970635	0.048387	**17**	0.520077	0.692935	0.561615	0.607527
**3**	0.97	0.138587	0.959098	0.086022	**18**	0.49	0.717391	0.527006	0.639785
**4**	0.948845	0.179348	0.92344	0.123656	**19**	0.456271	0.741848	0.486628	0.672043
**5**	0.923267	0.25	0.902465	0.177419	**20**	0.417492	0.76087	0.445726	0.698925
**6**	0.90	0.288043	0.893026	0.236559	**21**	0.39	0.796196	0.414263	0.741935
**7**	0.857261	0.347826	0.866807	0.274194	**22**	0.346535	0.82337	0.384373	0.774194
**8**	0.827008	0.388587	0.832722	0.311828	**23**	0.309131	0.847826	0.341898	0.801075
**9**	0.80	0.418478	0.804405	0.354839	**24**	0.27	0.861413	0.295752	0.833333
**10**	0.766502	0.453804	0.782905	0.392473	**25**	0.240374	0.894022	0.263765	0.865591
**11**	0.735974	0.491848	0.745149	0.419355	**26**	0.19967	0.918478	0.205558	0.892473
**12**	0.70	0.529891	0.717881	0.451613	**27**	0.15	0.942935	0.147352	0.913978
**13**	0.662266	0.557065	0.681699	0.483871	**28**	0.108361	0.964674	0.106974	0.946237
**14**	0.626238	0.597826	0.643419	0.510753	**29**	0.05308	0.98913	0.045097	0.973118
**15**	0.59	0.630435	0.613529	0.548387	**30**	0.00	1	0	1

In **Table 2**, SA [35], MSL [36], EP [37], PS [37], PSO [5] and MODE are taken to compare those obtained results for minimum fuel cost, minimum emission rate and compromise results. In comparison to other alternatives for solving this five-unit problem, it can be found that AGB-MOCDE can obtain better minimum fuel cost and minimum emission rate than other methods, and obtained compromise scheme dominates all optimal schemes by other alternatives, which means that AGB-MOCDE can properly optimize fuel cost and emission rate simultaneously for solving DEED problem, and it can also retain extreme objective value for completely analysis on obtained Pareto front.

**Table 2:** The comparisons among different optimization methods.

Methods	Minimum cost		Minimum emission		Compromise result	
	Cost($)	Emission(lb)	Cost($)	Emission(lb)	Cost($)	Emission(lb)
SA[35]	47356	**-**	**-**	**-**	**-**	**-**
MSL[36]	49216.81	**-**	**-**	**-**	**-**	**-**
EP[37]	46777	**-**	**-**	**-**	**-**	**-**
PS[37]	46530	**-**	**-**	**-**	47911	18927
PSO[5]	47852	22405	**-**	**-**	50893	20163
MODE	46934	18194	48841	18008	47714	18084
AGB-MOCDE	**46230**	**18295**	**49866**	**17927**	**47589**	**18075**

**Fig 4:**
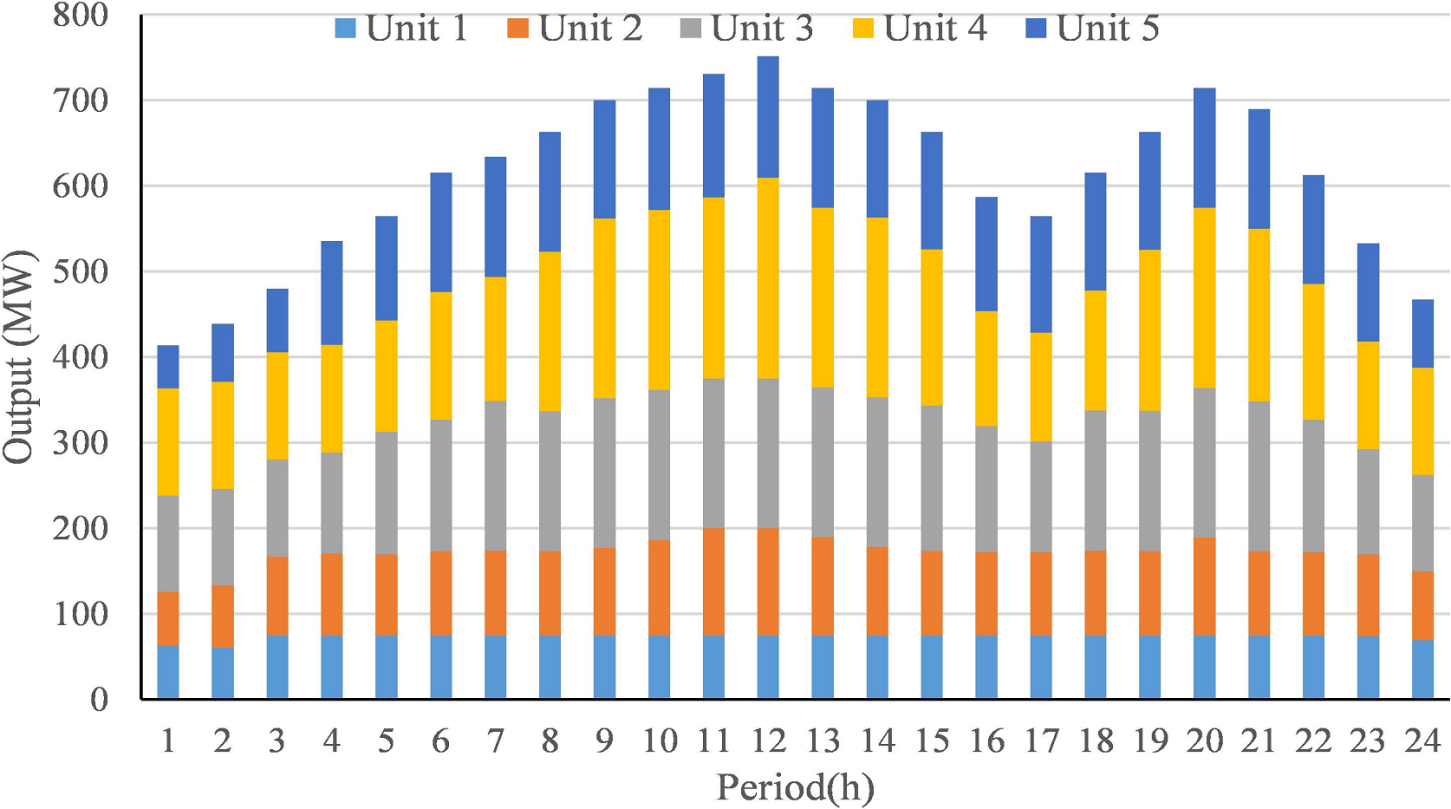
The output process of compromise scheme by AGB-MOCDE in test system 1.

Then, further analysis is taken on the output process of compromise scheme obtained by AGB-MOCDE, the output process is shown in **Fig 4**. The output of each thermal unit at each time period can properly satisfy those constraint limits, and system load balance at each time period is also properly satisfied with considering transmission loss of five thermal-unit, which has been presented in **Table 3**. It can be seen that transmission loss period can’t exceed 2% of system load at each time, and nonlinear transmission loss has been properly controlled within permitted accuracy.

**Table 3:** The transmission loss of five thermal-unit system.

Period(h)	1	2	3	4	5	6	7	8	9	10	11	12
Loss (MW)	3.508	3.925	4.715	5.822	6.424	7.63	8.078	8.869	9.922	10.341	10.869	11.554
Period(h)	13	14	15	16	17	18	19	20	21	22	23	24
Loss (MW)	10.36	9.927	8.857	6.941	6.437	7.62	8.869	10.361	9.608	7.565	5.748	4.445

According to those obtained results in this test system, the proposed AGB-MOCDE can properly solve DEED problem of five-thermal-unit power system with satisfying those constraint limits. The comparison with MODE reveals that adaptive grid mechanism can improve the diversity distribution of Pareto front, and the comparison with other alternatives on fuel cost and emission rate demonstrates that Cauchy mutation operator can promotes the optimal efficiency of differential evolution. After checking those constraint limits, all the constraint limits are properly satisfied and transmission loss is also controlled in permitted accuracy. Combined with above results, it can be found that the proposed AGB-MOCDE can be taken as a viable alternative for solving DEED problem.

The main parameter settings in this test system are presented as follows: The population size is set to 60, the size of archive set *N*_*Q*_ is 30, and maximum generation number *G*_max_ is 1000. Since wind power is not taken into consideration, the scenarios method is not used in this test system. The permitted total violation accuracy is set to 0.1, and the permitted output violation is set to 0.01, and coarse adjustment number is set to 5, and fining tuning number is set to 10. The reference membership value *μ*_*rq*_ is set to 0.75, and the initial square length $\delta_{F_{1}},\delta_{F_{2}}$ represents the square length are set to 200 and 20.

### 2. Test system 2

**Fig 5:**
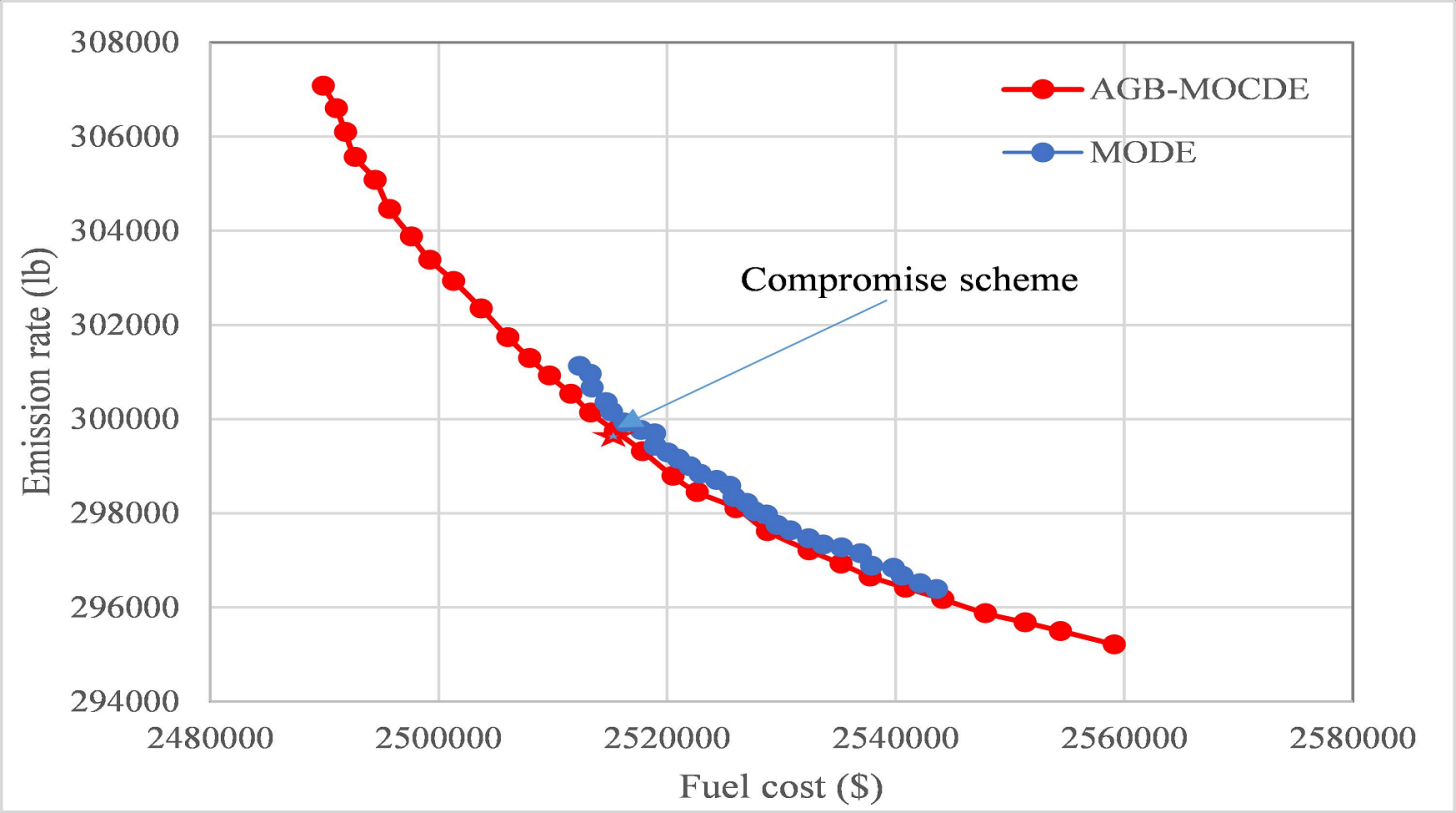
Pareto front obtained by AGB-MOCDE and MODE.

In this test system, thermal power output is taken as a decision variable, its main goal is to minimize the fuel cost and emission rate simultaneously, and all details on the data can be found in literature [14]. After the proposed AGB-MOCDE is implemented for the thermal power system, the Pareto front and membership value of non-dominated schemes can be properly obtained, as shown in **Fig 5** and **Table 4**. The Pareto front is obtained using 30 non-dominated schemes; all schemes are evenly distributed on the Pareto front. In comparison to MODE, AGB-MOCDE has better results, while all non-dominated schemes have a wider extreme edge and better diversity distribution.

**Table 4:** The membership of non-dominated schemes between AGB-MOCDE and MODE for test system 2.

Scheme	AGB-MOCDE		MODE		Scheme	AGB-MOCDE		MODE	
	Cost	Emission	Cost	Emission		Cost	Emission	Cost	Emission
**1**	1	0	1	0	**16**	0.630515	0.617952	0.567317	0.587392
**2**	0.983555	0.040034	0.970736	0.03521	**17**	0.596208	0.654109	0.531809	0.613747
**3**	0.972081	0.083102	0.965325	0.096774	**18**	0.55797	0.698525	0.511286	0.650221
**4**	0.95971	0.127518	0.925399	0.161923	**19**	0.527132	0.727181	0.476483	0.66498
**5**	0.934262	0.16831	0.910639	0.205355	**20**	0.477868	0.756426	0.447187	0.711786
**6**	0.916256	0.220565	0.874268	0.253637	**21**	0.438489	0.796966	0.410207	0.737086
**7**	0.888727	0.269954	0.828259	0.288003	**22**	0.385844	0.831606	0.358947	0.772296
**8**	0.865027	0.311504	0.789998	0.301919	**23**	0.345467	0.855373	0.318029	0.799916
**9**	0.835171	0.349178	0.789037	0.35758	**24**	0.308343	0.87855	0.266481	0.812144
**10**	0.800243	0.398399	0.754074	0.386886	**25**	0.263486	0.898441	0.214036	0.839764
**11**	0.766601	0.44981	0.72417	0.415138	**26**	0.216679	0.91909	0.182755	0.894371
**12**	0.73936	0.487063	0.690552	0.449926	**27**	0.162849	0.944037	0.120546	0.903647
**13**	0.714302	0.518837	0.662536	0.48366	**28**	0.112198	0.960725	0.096885	0.939279
**14**	0.686845	0.551117	0.617136	0.511701	**29**	0.067819	0.975811	0.045945	0.973013
**15**	0.662235	0.584829	0.580508	0.537634	**30**	0	1	0	1

According to the 30 obtained non-dominated schemes in **Fig 5**, the minimum cost of AGB-MOCDE is $22437 less than that of MODE, and the minimum emission rate of AGB-MOCDE is 1172 lb less than that of MODE. Among the 30 non-dominated schemes, approximately 20 AGB-MOCDE schemes have a lower fuel cost and a higher emission rate than those of MODE; the obtained results reveal that AGB-MOCDE has higher efficiency than MODE when solving the DEED problem.

For further analysis of the output process of the thermal units, a compromise scheme can be selected from the non-dominated schemes with the fuzzy satisfying method. The reference value *μ*_*rq*_ is set to 0.75; it can then be calculated that scheme (16) of AGB-MOCDE is taken as the compromise scheme and scheme (16) of MODE is taken as the compromise scheme. In comparison to different results obtained in literature [14] and literature [38], it can be found in **Table 5** that AGB-MOCDE has both lower minimum fuel cost and minimum emission rate and also has a relatively good compromise scheme. AGB-MOCDE integrates the Cauchy mutation operation into the MODE, which needs to verify the population diversity and calculate the convergence metric. The computational time is longer than that for MODE but still shorter than those for the alternatives in literature [14] and literature [38].

**Table 5:** The comparison among different optimization methods for the DEED problem.

Methods	Minimum cost		Minimum emission		Compromise result		Time(s)
	Cost ($)	Emission (lb)	Cost ($)	Emission (lb)	Cost ($)	Emission (lb)	
NSGA-II [14]	-	**-**	**-**	**-**	2522600	309940	20 min 11.475 s
RCGA [14]	2516800	317400	2656300	304120	2525100	312460	Approximately 18 min
MADM in [38]	-	-	**-**	**-**	2590500	291960	Approximately 15 min
MODE	2512327	301130	2543560	296387	2525841	298344	16 min 81 s
**AGB-MOCDE**	**2489890**	**307080**	**2559089**	**295215**	**2515458**	**299748**	**17 min 56 s**

The output process of the compromise scheme of AGB-MOCDE is presented in **Table 6**. All the output of thermal units is properly controlled in the feasible domain; the output limits, ramp rate limits and system load balance are properly satisfied in each time period. According to calculation of the transmission loss in each time period, the transmission loss does not exceed 5% of the system load, which reveals that transmission loss is also controlled properly. Thermal units 8 and 9 maintain the maximum output due to their low power capacity, while thermal units 5, 6 and 7 almost maintain the maximum output during the entire time period.

**Table 6:** The output (MW) details of the compromise scheme obtained by AGB-MOCDE for test system 2.

Hours	P_1_	P_2_	P_3_	P_4_	P_5_	P_6_	P_7_	P_8_	P_9_	P_10_	Load	Loss
**1**	150	135	92.331	120.418	125.613	126.806	94.844	119.972	80	10.568	1036	19.552
**2**	150	135	91.591	114.86	169.587	123.369	124.844	119.948	80	23.216	1110	22.415
**3**	150	135	161.543	127.767	179.443	159.963	130	120	80	42.756	1258	28.472
**4**	150.767	169.165	185.031	177.581	228.563	159.968	130	120	80	40.635	1406	35.71
**5**	150	211.558	185.403	186.489	241.677	159.94	129.934	120	80	55	1480	40.001
**6**	211.865	222.138	218.9	236.489	243	160	129.698	120	80	54.999	1628	49.089
**7**	227.13	227.219	267.549	247.012	242.815	159.951	130	120	80	54.229	1702	53.905
**8**	231.151	275.244	285.047	255.876	243	160	130	120	80	55	1776	59.318
**9**	299.976	305.047	302.395	299.648	243	160	130	120	80	54.975	1924	71.041
**10**	330.343	343.681	339.927	299.941	243	160	130	120	80	54.672	2022	79.564
**11**	378.274	387.934	339.764	300	243	159.977	130	120	79.994	54.946	2106	87.889
**12**	400.338	414.195	340	300	243	160	129.997	120	79.94	55	2150	92.47
**13**	362.854	366.227	339.402	300	243	160	129.984	120	79.997	55	2072	84.464
**14**	301.021	309.819	299.354	299.701	243	160	130	120	80	52.206	1924	71.101
**15**	235.411	277.106	281.021	253.852	243	160	130	120	80	55	1776	59.39
**16**	176.03	222.053	201.021	233.25	243	160	129.923	120	79.947	33.119	1554	44.343
**17**	152.101	219.774	187.965	183.25	243	160	130	119.966	80	44.017	1480	40.073
**18**	221.593	222.673	212.131	232.889	242.978	160	129.959	120	80	55	1628	49.223
**19**	227.652	253.164	286.044	280.158	243	160	130	120	80	54.992	1776	59.01
**20**	304.889	322.128	331.962	299.988	243	159.99	130	120	80	55	1972	74.957
**21**	303.523	302.87	310.941	289.745	243	160	130	120	80	55	1924	71.079
**22**	223.523	222.87	231.582	239.745	235.061	160	129.642	120	80	34.757	1628	49.18
**23**	150	142.898	151.582	189.745	212.161	160	130	120	80	27.451	1332	31.837
**24**	150	135	108.117	139.745	168.336	158.707	129.162	119.989	80	20.188	1184	25.244

The main parameter settings are presented as follows: The size of archive set is set to 30, size of evolutionary population is set to 100, and maximum generation number *G*_max_ is 1000, permitted constraint violation accuracy of output is set to 0.01MW, permitted total violation is set to 0.1MW, threshold parameter *ξ*_*CP*_ is set to 0.04, crossover rate is set to 0.3. According to the above comparison and analysis, it can be found that the proposed AGB-MOCDE has both better convergence ability and diversity distribution when solving the DEED problem. In comparison to MODE and other alternatives, AGB-MOCDE can produce a set of non-dominated schemes that have a wider extreme edge and a more evenly distributed diversity distribution. In the output process, the output of each thermal unit at each time period is properly controlled within the feasible domain; system load balance with transmission loss is also properly dealt with using the proposed constraint handling technique.

### 3. Test system 3

**Fig 6:**
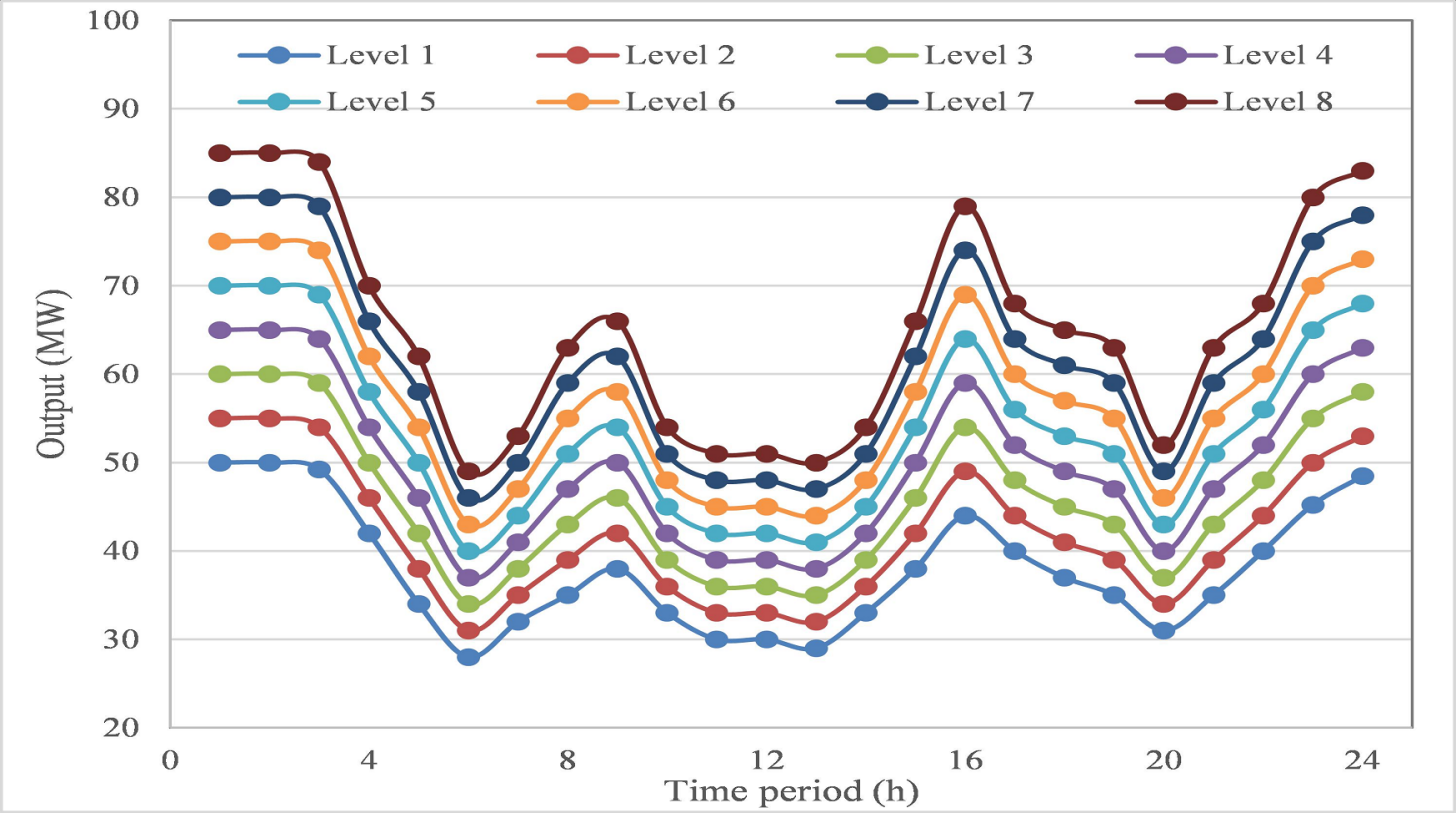
Interval division of output domain for wind farm #1.

**Fig 7:**
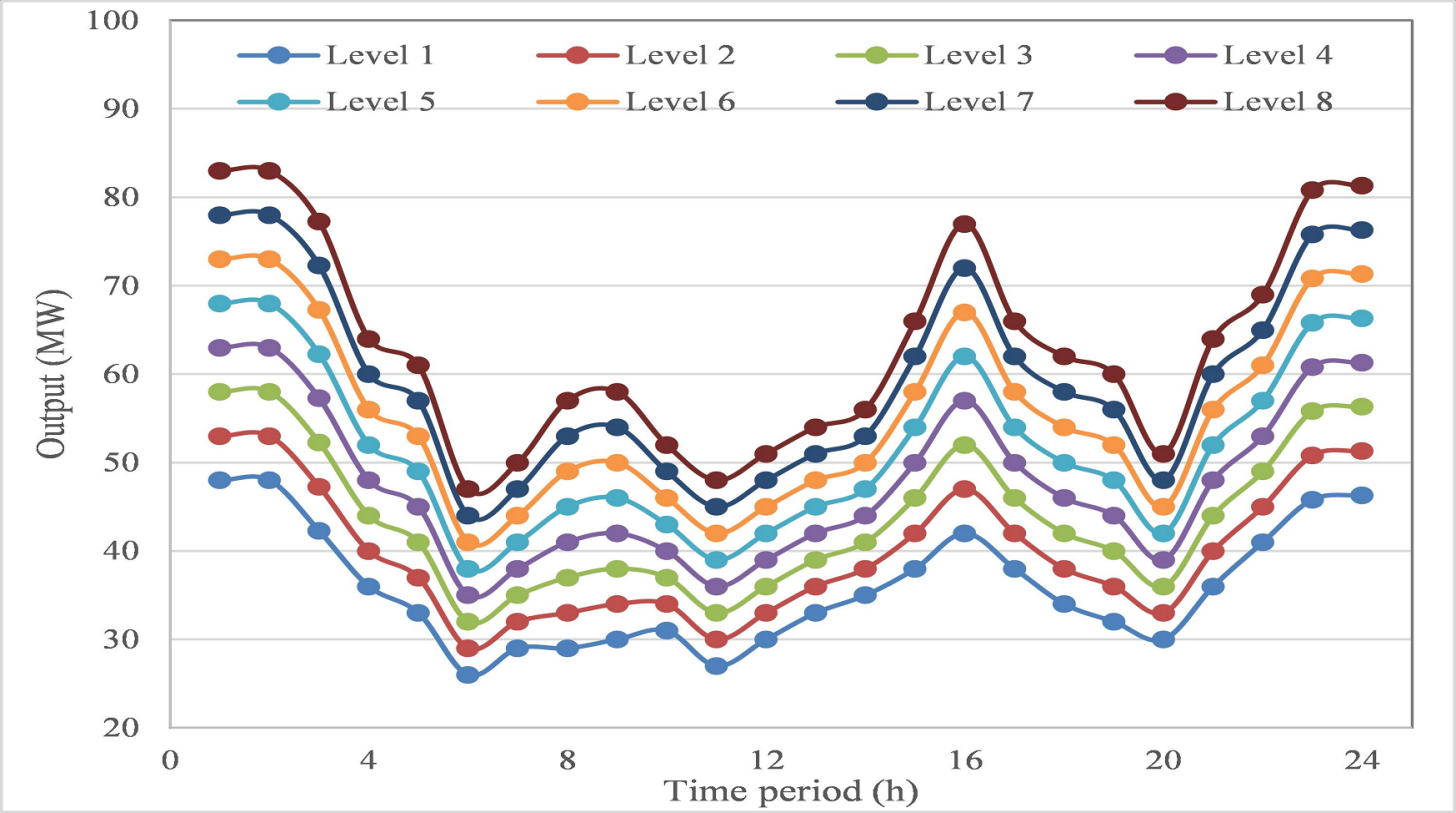
Interval division of output domain for wind farm #2.

**Fig 8:**
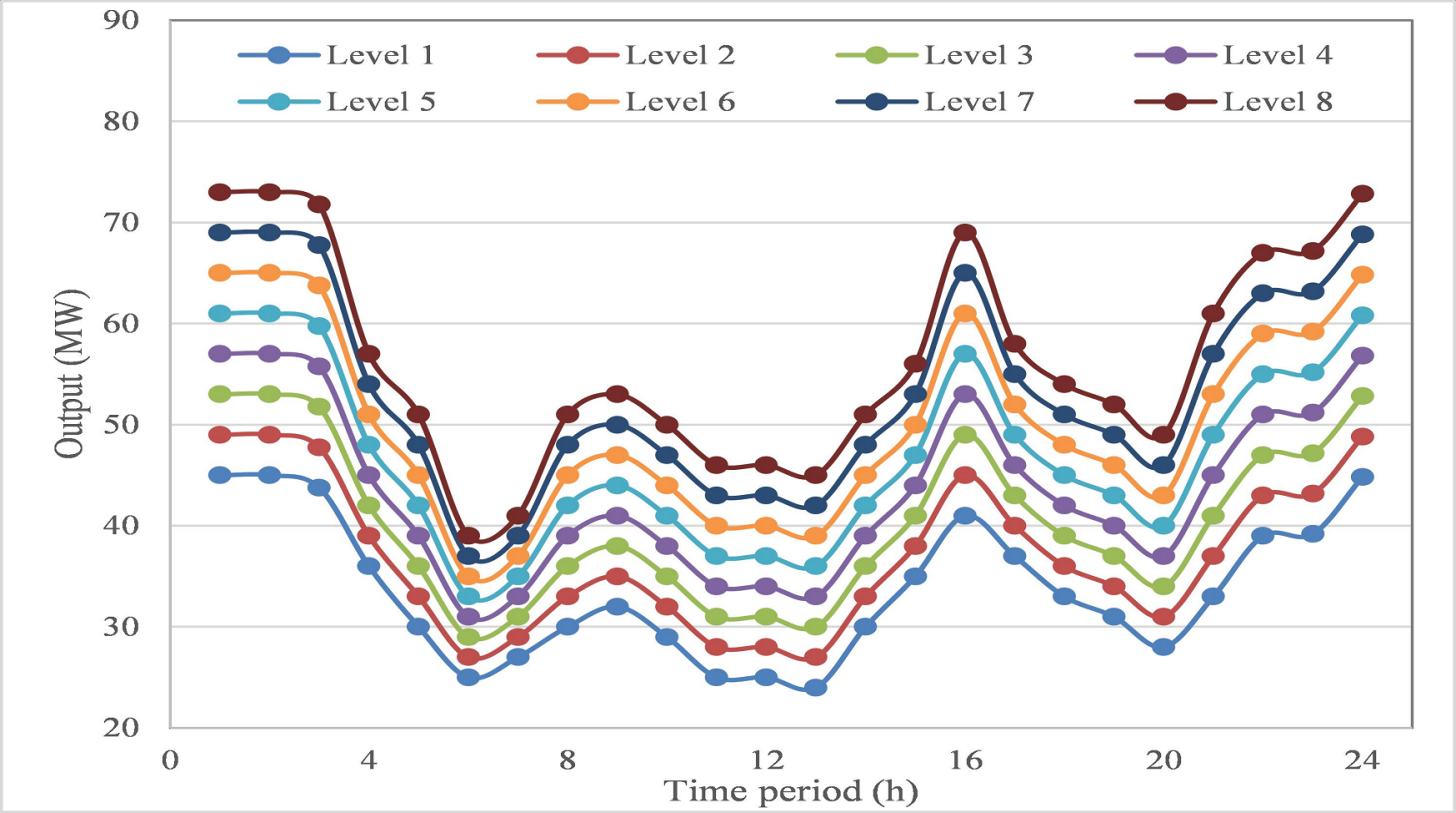
Interval division of output domain for wind farm #3.

Since wind power is integrated into the DEED problem, the scenario-based method is utilized to simulate those possible outputs of wind power generation. The uncertainty domain of wind power in each time period is equally divided into several levels, which represent those possible situations caused by wind power generation. In this test system, the uncertainty domain of wind power output in three wind farms is divided into 7 intervals with 8 levels, which are shown in **Figs 6–8**. Among these divided intervals, interval 1 represents extreme case 1 of the worst situation for wind power generation, while interval 7 represents extreme case 2 of the best situation for wind power generation. Combined with the probability density function of wind power generation, the probability of each interval can be calculated using formulation (25), and the probability of the generated scenario can be calculated using formulation (26).

**Fig 9:**
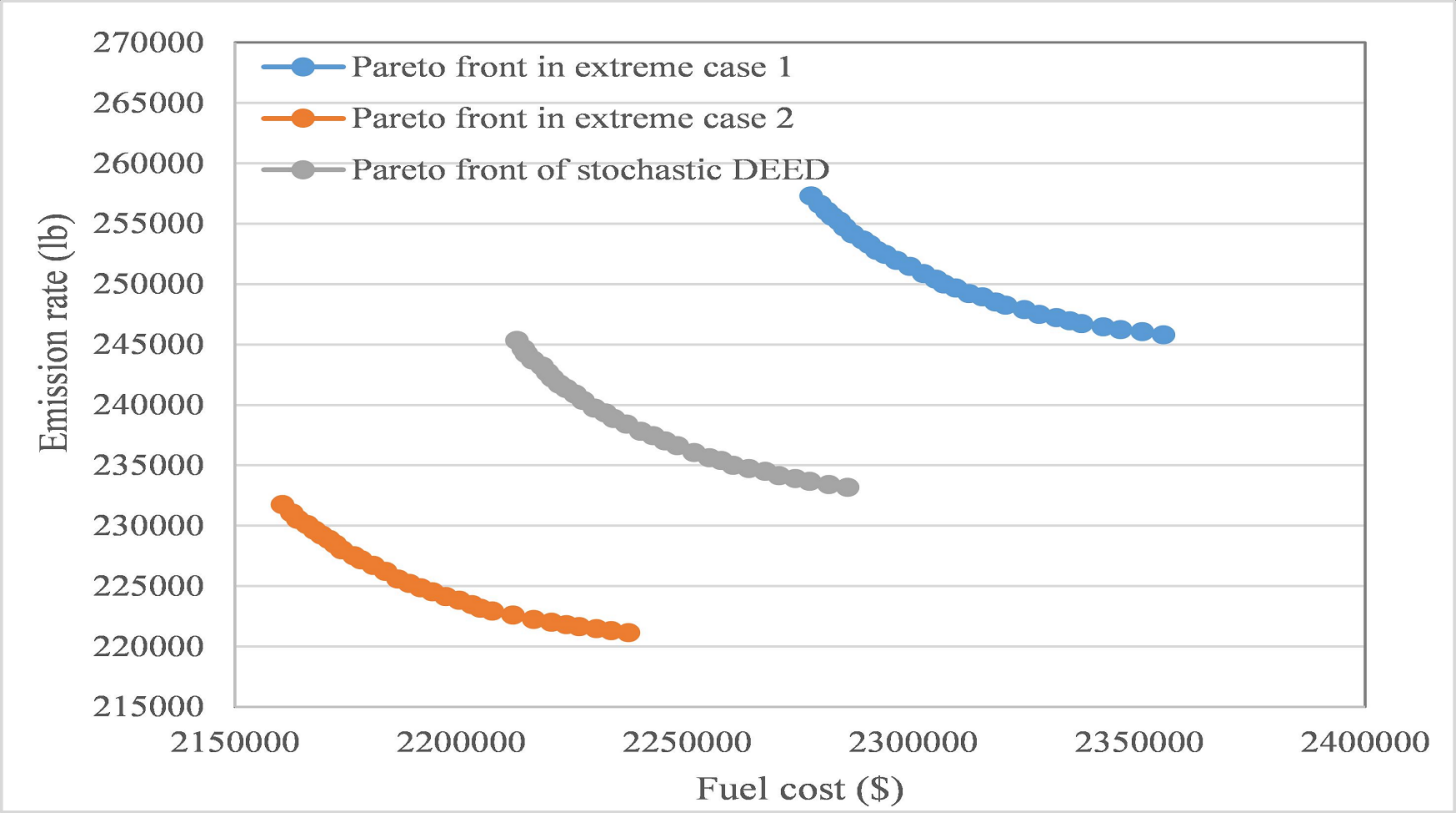
The obtained optimal schemes in two extreme cases and in stochastic DEED.

According to different intervals of wind power output, the proposed AGB-MOCDE is implemented on thermal-wind power system with optimizing fuel cost and emission rate simultaneously, and 30 non-dominated schemes can be properly obtained in the archive set. Extreme case 1 and extreme case 2 are taken for comparison with the Pareto front of stochastic DEED, the obtained Pareto fronts are shown in **Fig 9**. Since less wind power generation occurs in extreme case 1, more thermal power generation is needed to meet the system load requirement, which also leads more fuel cost and emission rate. Similarly, case 3 has less fuel cost and emission rate due to more wind power generation. The stochastic DEED is the situation between these two extreme cases, the obtained fuel cost and emission rate are range in the interval between these two extreme cases, which can be seen in **Fig 9**.

In extreme case 1, wind power output occurs in the poorest situation of wind resources, which is also the worst case in the thermal-wind power system. After fuzzy satisfying method is utilized to select a compromise scheme from the above non-dominated schemes, the thermal output details of the compromise scheme are as shown in **Table 7**. It can be observed that the output of each thermal unit in each time period is properly controlled in the feasible domain, while output limits, ramp rate limits and system load balance are properly handled. The transmission loss in each time period cannot exceed 5% of the system load requirement, which also reveals that system load balance is properly dealt with by the transmission loss among thermal units and wind farms.

**Table 7:** The output (MW) details of the optimal scheme of AGB-MOCDE in extreme case 1.

Hours	P_1_	P_2_	P_3_	P_4_	P_5_	P_6_	P_7_	P_8_	P_9_	P_10_	Loss
**1**	150	135	73	61.431	120.722	116.867	92.978	85.309	52.439	12.821	14.567
**2**	150	135	82.891	84.393	122.948	122.479	93.438	86.089	80	19.72	16.958
**3**	150	135	92.785	115.762	168.329	125.505	123.438	116.089	80	31.587	22.645
**4**	150	135.473	149.165	132.418	218.329	159.936	130	120	80	40.986	29.807
**5**	150	161.56	183.926	180.425	223.284	159.965	130	120	79.977	22.531	34.168
**6**	210.38	222.289	196.015	192.059	243	160	129.963	119.997	80	35.539	44.242
**7**	222.577	222.205	206.557	218.873	242.994	160	130	120	79.978	54.981	48.165
**8**	222.235	222.149	255.219	241.318	242.802	160	129.967	120	80	54.998	52.188
**9**	252.587	288.472	289.374	262.72	243	160	130	120	79.982	55	62.635
**10**	302.686	309.58	295.395	300	243	160	130	120	80	55	71.161
**11**	327.14	343.668	340	300	243	160	130	120	80	55	79.308
**12**	360.044	364.267	340	300	243	160	129.787	120	80	46.834	83.432
**13**	316.189	324.677	328.542	300	243	160	130	120	80	54.987	75.895
**14**	248.885	287.337	284.237	275.921	243	160	130	120	80	54.881	62.761
**15**	221.088	222.466	238.156	241.106	243	160	130	120	80	54.798	51.114
**16**	151.217	183.569	183.966	191.106	223.338	159.945	130	120	80	33.353	36.494
**17**	150.127	142.502	174.846	180.899	222.514	159.981	129.875	120	80	31.917	33.161
**18**	183.43	222.188	195.498	187.773	243	160	130	119.979	80	39.102	42.47
**19**	228.118	234.457	251.985	237.773	243	160	130	120	80	39.264	52.097
**20**	283.229	304.438	284.392	285.9	243	160	130	120	80	54.995	67.454
**21**	281.404	270.03	282.918	254.177	243	160	130	120	80	54.99	62.519
**22**	201.404	190.03	202.918	204.177	205.837	160	130	120	80	49.058	41.424
**23**	150	135	124.093	154.177	172.898	127.353	129.59	120	80	27.531	25.757
**24**	150	135	82.599	105.363	122.898	122.487	126.656	120	79.969	12.331	19.678

In extreme case 2, wind farms make full use of their wind turbines when the wind speed is at the maximum level. Since system load requirement in each time period is certain, the thermal units bear relative low output in comparison to other cases, and thermal output details of the compromise scheme are shown in **Table 8**. The output of each thermal unit in each time period is properly controlled in the feasible domain, and system load balance at each time period is properly satisfied. In comparison to those obtained results in extreme case 1, thermal output and transmission loss are smaller than that in extreme case 1 at most time.

**Table 8:** The output (MW) details of the optimal scheme of AGB-MOCDE in extreme case 2.

Hours	P_1_	P_2_	P_3_	P_4_	P_5_	P_6_	P_7_	P_8_	P_9_	P_10_	Loss
**1**	150	135	73	60	73.8	115.189	57.077	85.305	52.065	12.682	12.118
**2**	150	135	73	62.019	121.692	113.872	87.077	85.185	52.408	10	14.253
**3**	150	135	95.205	108.078	128.359	130.935	96.342	115.179	80	12.267	19.405
**4**	150	135.925	134.805	125.194	175.076	160	126.342	120	80	40.003	26.845
**5**	150	140.639	153.116	146.157	222.484	160	129.951	120	80	40.116	30.963
**6**	164.415	219.415	188.561	180.845	243	160	130	120	80	51.893	41.129
**7**	216.912	221.972	190.617	189.872	242.98	160	130	120	80	55	45.353
**8**	222.432	222.628	205.607	220.188	242.849	160	130	120	80	55	48.204
**9**	229.338	268.755	279.343	247.332	243	160	130	120	80	52.399	57.667
**10**	283.838	302.07	286.429	277.039	243	160	130	120	80	54.993	66.869
**11**	307.331	312.506	332.043	300	243	160	130	120	80	55	74.38
**12**	329.394	327.206	340	300	242.999	160	130	120	80	55	78.099
**13**	304.245	309.834	308.777	300	243	160	129.967	120	79.885	43.196	71.404
**14**	243.582	282.195	260.798	252.157	243	159.989	130	120	80	54.755	58.976
**15**	217.928	222.291	195.048	227.823	243	159.964	130	119.99	80	44.566	47.11
**16**	150	142.943	160.615	177.823	222.154	160	129.975	120	80	24.524	32.034
**17**	150	135.42	150.45	147.02	222.221	160	130	120	79.964	28.491	30.066
**18**	153.272	209.292	185.01	180.665	225.166	160	130	120	79.97	47.626	38.501
**19**	220.343	222.32	204.374	225.652	237.814	160	130	119.938	80	53.979	47.92
**20**	238.226	301.395	284.374	275.652	243	160	130	120	80	54.873	63.02
**21**	241.919	263.599	269.162	236.396	243	160	130	120	80	55	57.076
**22**	161.919	184.823	189.162	186.396	221.248	160	129.91	119.118	80	34.557	37.133
**23**	150	135	109.162	136.396	171.248	122.454	122.461	89.118	71.423	26.088	22.35
**24**	150	135	80.644	90.861	122.9	122.521	92.988	85.667	80	10	16.721

Combined with the probabilities of different output intervals, the stochastic DEED model in Section 2 can be optimized by using AGB-MOCDE, the obtained Pareto front is shown in **Fig 9**. After these scenarios are generated in this test system, obtained optimal schemes can be taken as best schemes for most possibilities. The compromise scheme can be properly selected from archive set with fuzzy satisfying method, the output details of compromise scheme are shown in **Table 9**. In comparison to the above two extreme cases, the thermal output is within thermal output interval of extreme case 1 and extreme case 2, transmission loss can also be controlled properly, and thermal output at each time period satisfies all the constraint limits. In a real-world application, compromise scheme in stochastic DEED can be taken as the optimal scheme for guiding the output assignment in the thermal-wind power system. The main parameter settings in this test system are presented as follows: The size of archive set is set to 30, size of evolutionary population is set to 100, and maximum generation number *G*_max_ is 1000, permitted constraint violation accuracy of output is set to 0.01MW, permitted total violation is set to 0.1MW, threshold parameter *ξ*_*CP*_ is set to 0.05, crossover rate is set to 0.4, and scenarios number is set to 50.

**Table 9:** The output (MW) details of the optimal scheme of AGB-MOCDE in stochastic DEED.

Hours	P_1_	P_2_	P_3_	P_4_	P_5_	P_6_	P_7_	P_8_	P_9_	P_10_	Loss
**1**	150	135	73	60	89.641	109.167	92.743	85.172	52.584	10	13.307
**2**	150	135	73	67.334	121.323	118.681	93	85.292	80	10	15.63
**3**	150	135	88.485	106.901	163.133	122.729	122.69	115.292	80	10.746	21.016
**4**	150	135	132.949	121.499	212.768	159.987	129.666	119.98	80	40	28.349
**5**	150	139.993	166.871	167.84	222.776	159.989	130	120	80	39.5	32.469
**6**	188.112	219.993	195.554	183.347	242.928	160	130	120	80	43.718	42.652
**7**	180.142	221.971	209.116	233.075	243	159.974	130	119.985	80	55	46.263
**8**	219.129	222.125	224.712	239.671	243	159.978	130	120	80	54.994	50.109
**9**	251.941	281.676	272.313	253.328	243	160	129.999	120	79.987	53.537	60.281
**10**	295.115	304.55	292.316	286.522	243	160	130	120	80	54.998	69.001
**11**	315.875	325.84	339.634	300	243	160	130	119.927	80	55	76.776
**12**	337.938	348.416	340	300	243	159.925	130	120	79.897	55	80.676
**13**	306.123	310.73	323.446	299.989	243	160	129.936	120	80	54.773	73.497
**14**	228.737	282.292	285.121	271.064	243	160	130	120	80	54.89	60.604
**15**	226.173	222.082	218.081	229.34	242.98	160	129.889	120	80	47.114	49.159
**16**	150	151.219	182.367	180.628	222.768	160	129.919	120	80	35.244	34.145
**17**	150	135.459	161.816	171.547	222.633	159.987	129.92	120	80	26.688	31.55
**18**	158.224	215.183	191.675	186.702	243	160	130	120	80	41.074	40.358
**19**	224.892	221.441	218.445	236.665	243	160	130	120	80	54.997	49.94
**20**	260.367	294.678	288.416	285.13	243	160	130	120	80	55	65.091
**21**	230.073	294.358	281.275	243.979	243	160	130	120	80	55	59.685
**22**	150.073	214.358	201.275	193.979	222.349	159.911	130	119.845	80	33.391	39.181
**23**	150	135	121.275	143.979	172.364	125.668	111.79	120	80	16.938	24.014
**24**	150.001	135	75.884	96.283	122.998	122.744	93.097	120	80	18.069	18.216

DEED with wind uncertainty in different intervals can represent possible situations in a real-world application, the optimal scheme in extreme case 1 can guide the DEED, especially when wind power is generated under the weakest wind speed at the three wind farms, and the optimal scheme in extreme case 2 can deal with the situation in which wind power is generated under abundant wind resources for power generation. The optimal schemes obtained for the above two extreme cases may cause the conservation problem in the stochastic DEED problem, the proposed AGB-MOCDE with scenario-based method generates the most probable optimal scheme with considering probability density function of wind power generation, which can be considered the optimal scheme that can better fit real-world application.

## Conclusions

Since wind power generation is taken into consideration in the DEED problem, stochastic characteristics of wind power brings a great challenge for optimizing stochastic DEED problem. For properly tackling with above problem, this paper proposes an adaptive grid-based multi-objective Cauchy differential evolution combining with scenario-based technique to solve the DEED problem. The improved scenario-based technique simulates those possible situations according to different levels of stochastic domain, which divides the output domain of the wind power into several intervals. In comparison to other alternatives for solving DEED problem, the proposed method has following innovations: (1) For properly improving convergence ability and diversity distribution of Pareto front, an adaptive grid-based mechanism and Cauchy mutation operator are interpolated into multi-objective differential evolution, which mainly manipulates Pareto-optimal solution with grid-based technique and improves optimization efficiency with considering population diversity; (2) For reducing computational complexity of generated scenarios, reduction mechanism of scenario-based method is improved with covariance among different scenarios; (3) Since emission rate and economic cost are taken into consideration simultaneously, Pareto dominance-based multi-objective evolutionary algorithm is utilized to optimize two objectives in single optimization run. In this mode, a set of Pareto optimal schemes are produced instead of single optimal solution, which can provide more viable choices for decision makers. In three test systems, the obtained optimal schemes represent different possible situations in DEED, the conservation of the obtained optimal scheme can be decreased according to the probability density function, and the compromise scheme of stochastic DEED can be taken as the optimal scheme in real-world applications.

## Supporting information

S1 Table Data for Fig 4. (PDF)

S2 Table Wind power generation. (PDF)
