# Supplementary material for: Adaptive grid based multi-objective Cauchy differential evolution for stochastic dynamic economic emission dispatch with wind power uncertainty
Source: PLoS One. 2017 Sep 29;12(9):e0185454. doi: 10.1371/journal.pone.0185454 (PMC5621695; doi:10.1371/journal.pone.0185454)
Supplement: S1 Table — (PDF) [file pone.0185454.s001.pdf]

	Unit 1	Unit 2	Unit 3	Unit 4	Unit 5
1	62.989	62.689	112.941	124.889	50
2	60.907	72.462	112.758	124.872	67.926
3	74.875	92.051	113.695	124.807	74.287
4	74.992	95.719	117.926	125.408	121.777
5	75	94.787	143.159	129.724	121.754
6	75	98.57	153.254	149.157	139.649
7	75	99.066	174.472	145.054	140.486
8	74.942	98.606	162.945	186.444	139.932
9	74.931	102.199	174.964	209.813	138.015
10	75	111.609	174.983	209.993	142.756
11	75	124.891	175	211.553	144.425
12	75	124.895	175	234.466	142.193
13	75	114.473	174.978	210.183	139.726
14	75	103.535	174.626	209.823	136.943
15	75	98.475	169.588	182.796	136.998
16	74.908	97.373	147.073	134.132	133.455
17	75	97.291	129.485	126.605	136.056
18	74.986	98.698	164.065	139.908	137.963
19	75	98.255	164.279	187.452	137.883
20	75	114.123	174.949	210.215	140.074
21	74.934	98.471	174.978	201.537	139.688
22	75	97.216	154.872	158.013	127.464
23	74.066	95.516	122.87	125.435	114.861
24	69.316	80.473	112.646	124.976	80.034
