# Supplementary material for: Adaptive grid based multi-objective Cauchy differential evolution for stochastic dynamic economic emission dispatch with wind power uncertainty
Source: PLoS One. 2017 Sep 29;12(9):e0185454. doi: 10.1371/journal.pone.0185454 (PMC5621695; doi:10.1371/journal.pone.0185454)
Supplement: S2 Table — (PDF) [file pone.0185454.s002.pdf]

	Level 1			Level 2			Level 3	
	Wind #1	Wind #2	Wind #3	Wind #1	Wind #2	Wind #3	Wind #1	Wind #2
1	50	48	45	55	53	49	105	101
2	50	48	45	55	53	49	105	101
3	49.22	42.28	43.76	54	47.28	47.76	103.22	89.56
4	42	36	36	46	40	39	88	76
5	34	33	30	38	37	33	72	70
6	28	26	25	31	29	27	59	55
7	32	29	27	35	32	29	67	61
8	35	29	30	39	33	33	74	62
9	38	30	32	42	34	35	80	64
10	33	31	29	36	34	32	69	65
11	30	27	25	33	30	28	63	57
12	30	30	25	33	33	28	63	63
13	29	33	24	32	36	27	61	69
14	33	35	30	36	38	33	69	73
15	38	38	35	42	42	38	80	80
16	44	42	41	49	47	45	93	89
17	40	38	37	44	42	40	84	80
18	37	34	33	41	38	36	78	72
19	35	32	31	39	36	34	74	68
20	31	30	28	34	33	31	65	63
21	35	36	33	39	40	37	74	76
22	40	41	39	44	45	43	84	86
23	45.23	45.82	39.18	50	50.82	43.18	95.23	96.64
24	48.47	46.32	44.82	53	51.32	48.82	101.47	97.64

	level 4			Level 5			Level 6	
Wind #3	Wind #1	Wind #2	Wind #3	Wind #1	Wind #2	Wind #3	Wind #1	Wind #2
94	155	149	139	205	197	184	255	245
94	155	149	139	205	197	184	255	245
91.52	152.44	131.84	135.28	201.66	174.12	179.04	250.88	216.4
75	130	112	111	172	148	147	214	184
63	106	103	93	140	136	123	174	169
52	87	81	77	115	107	102	143	133
56	99	90	83	131	119	110	163	148
63	109	91	93	144	120	123	179	149
67	118	94	99	156	124	131	194	154
61	102	96	90	135	127	119	168	158
53	93	84	78	123	111	103	153	138
53	93	93	78	123	123	103	153	153
51	90	102	75	119	135	99	148	168
63	102	108	93	135	143	123	168	178
73	118	118	108	156	156	143	194	194
86	137	131	127	181	173	168	225	215
77	124	118	114	164	156	151	204	194
69	115	106	102	152	140	135	189	174
65	109	100	96	144	132	127	179	164
59	96	93	87	127	123	115	158	153
70	109	112	103	144	148	136	179	184
82	124	127	121	164	168	160	204	209
82.36	140.46	142.46	121.54	185.69	188.28	160.72	230.92	234.1
93.64	149.94	143.96	138.46	198.41	190.28	183.28	246.88	236.6

	Level 7			Level 8		
Wind #3	Wind #1	Wind #2	Wind #3	Wind #1	Wind #2	Wind #3
229	305	293	274	355	341	319
229	305	293	274	355	341	319
222.8	300.1	258.68	266.56	349.32	300.96	310.32
183	256	220	219	298	256	255
153	208	202	183	242	235	213
127	171	159	152	199	185	177
137	195	177	164	227	206	191
153	214	178	183	249	207	213
163	232	184	195	270	214	227
148	201	189	177	234	220	206
128	183	165	153	213	192	178
128	183	183	153	213	213	178
123	177	201	147	206	234	171
153	201	213	183	234	248	213
178	232	232	213	270	270	248
209	269	257	250	313	299	291
188	244	232	225	284	270	262
168	226	208	201	263	242	234
158	214	196	189	249	228	220
143	189	183	171	220	213	199
169	214	220	202	249	256	235
199	244	250	238	284	291	277
199.9	276.15	279.92	239.08	321.38	325.74	278.26
228.1	295.35	282.92	272.92	343.82	329.24	317.74
